# Alcohol consumption is associated with glaucoma severity regardless of *ALDH2* polymorphism

**DOI:** 10.1038/s41598-020-74470-6

**Published:** 2020-10-15

**Authors:** Young Soo Han, Yong Woo Kim, Yu Jeong Kim, Ki Ho Park, Jin Wook Jeoung

**Affiliations:** 1 Department of Ophthalmology, Seoul National University Hospital, Seoul National University College of Medicine, 101 Daehak-ro, Jongno-gu, Seoul, 03080 Korea; 2 Department of Ophthalmology, Seoul National University Hospital, Seoul, Korea

**Keywords:** Clinical genetics, Genomics, Medical genetics, Population genetics, Medical research

## Abstract

The present study investigated the effect of aldehyde dehydrogenase2 (*ALDH2*) rs671 polymorphism and alcohol consumption on the severity of primary open-angle glaucoma (POAG). The questionnaire for alcohol consumption pattern and targeted genotyping for *ALDH2* rs671 polymorphism was performed from 445 Korean POAG patients. Retinal nerve fiber layer (RNFL) and ganglion cell-inner plexiform layer (GCIPL) thicknesses were measured and compared according to alcohol consumption and *ALDH2* rs671 genotype. Heavy drinking group eyes had thinner RNFL thickness than did abstinence group eyes (65.0 ± 10.9 vs. 70.9 ± 11.5 µm, *P* = 0.023). Both mild (65.8 ± 9.6 µm) and heavy (63.8 ± 8.4 µm) drinking group eyes had significantly thinner macular GCIPL thickness than did abstinence group eyes (68.1 ± 8.2 µm, *P* = 0.003). However, *ALDH2* rs671 polymorphism did not show any significant association with RNFL or GCIPL thickness. Alcohol consumption was significantly associated with GCIPL thinning (β = –0.446, *P* = 0.035) after adjustment for multiple confounding factors. As excessive alcohol consumption was significantly associated with thinner GCIPL thickness while *ALDH2* polymorphism had no significant effect on RNFL or GCIPL thickness, glaucoma patients should avoid excessive alcohol consumption regardless of *ALDH2* polymorphism.

## Introduction

Recent meta-analyses and large epidemiological studies have found that adult alcohol consumption and binge drinking are increasing worldwide^1–3^. Excessive alcohol consumption can cause not only alcohol-related disorders but also cardiovascular diseases^4^, cancers^5^, or neurodegenerative diseases such as Alzheimer’s disease^6^. However, it is debatable whether increased alcohol consumption is associated with development of glaucoma. Many population-based epidemiological studies as well as case–control studies have found no association between alcohol consumption and risk of glaucoma^7–12^. On the other hand, in the Framingham Eye Study, alcohol consumption was associated with prevalence of glaucoma^13^. The Black Women’s Health Study demonstrated that the risk of primary open-angle glaucoma (POAG) was greater in current alcohol consumers than in those who did not drink, and that this effect was stronger in women under 50^14^. A recent cross-sectional study showed that alcohol intake > 10 g/d for women and > 20 g/d for men was one of the significant factors associated with thinner peripapillary retinal nerve fiber layer (RNFL)^15^. Another large cross-sectional study, this one in the UK, also reported that frequent alcohol intake was associated with thinner macular RNFL, ganglion cell complex (GCC) and ganglion cell-inner plexiform layer (GCIPL) when compared with rare or no alcohol intake^16^.

Previous inconsistent findings regarding alcohol consumption and the risk of glaucoma may be attributed to individual differences in alcohol metabolism. Aldehyde dehydrogenase 2 (ALDH2) fulfills a crucial role in alcohol metabolism by degrading acetaldehyde to nontoxic acetic acid^17^. This enzyme is coded by the *ALDH2* gene, which is commonly polymorphic in East Asian populations^18,19^. A point mutation in the *ALDH2* gene (the rs671 allele) induces an inactive form of ALDH2 that results in reduced alcohol tolerance by the accumulation of acetaldehyde in the body^17^. People carrying the mutant rs671 allele show the characteristic acute effects of alcohol drinking, as mediated by acetaldehyde, such as facial flushing and increased pulse rate^20^. rs671 polymorphism is well established as being significantly associated with various chronic diseases such as cardiovascular disease, hypertension, cancer, and Alzheimer's disease^17^.

In this regard, it is worth investigating the specific relationship between rs671 polymorphism, alcohol consumption, and glaucoma severity. In this study, we evaluated peripapillary RNFL and macular GCIPL thickness in Korean POAG patients according to alcohol consumption and rs671 genotype.

## Results

### Subject demographics

Of the 445 POAG patients, 265 reported that they did not drink (the abstinence group) and another 180 that they drank (the drinking group). Of these 180 patients for whom alcohol consumption information was available, 147 were classified as mild drinkers and 33 as heavy drinkers. According to the binge drinking criteria, 141 patients were non-binge drinkers and 39 were binge drinkers. Patients in the drinking group were significantly younger (52.5 ± 13.6 years old) and comprised of more males (62.8%) than was the abstinence group (55.1 ± 12.8 years old and 40.4%, respectively, all *P* < 0.05). Although there was no significant difference in the prevalence of diabetes or hypertension between the two groups, the patients from the drinking group had significantly more smoking pack-years (2.8 ± 7.3 pack-years) than did the abstinence group (0.7 ± 3.3 pack-years, *P* < 0.001). The eyes from the drinking group were more myopic (–3.1 ± 3.8 vs. –2.1 ± 3.2 *D*, *P* = 0.003) and had greater AXL (25.1 ± 1.6 vs. 24.3 ± 1.5 mm, *P* < 0.001) than those from the abstinence group. The patients in the drinking group showed a greater proportion of the major allele (GG) for *ALDH2* rs671 polymorphism. Detailed information on the subject demographics is provided in Table 1.

**Table 1:** Subject demographics.

Variables	Abstinence group (*n* = 265)	Drinking group (*n* = 180)	*P* value
**Age**	**55.1 ± 12.8**	**52.5 ± 13.6**	0.038*
**Sex, female, n (%)**	**158 (59.6)**	**67 (37.2)**	**0.001**^†^
DM, *n* (%)	42 (15.8)	29 (16.1)	0.99^†^
HTN, *n* (%)	78 (29.4)	68 (37.8)	0.08^†^
Baseline IOP, mmHg	16.5 ± 5.0	17.1 ± 5.5	0.19*
**AXL, mm**	**24.29 ± 1.49**	**25.13 ± 1.59**	< 0.001*
**SE, D**	− **2.08 ± 3.24**	− **3.14 ± 3.76**	0.003*
CCT, μm	535.1 ± 31.3	536.8 ± 37.2	0.62*
**rs671 genotype, GG/GA/AA (%)**	**175(66.0)/79(29.8)/11(4.2)**	**147(81.7)/28(15.6)/5(2.8)**	**0.001**^†^
**Smoking, pack-years**	**0.7 ± 3.3**	**2.8 ± 7.3**	< 0.001*

Mean ± standard deviation, statistically significant values are shown in bold.

IOP, intraocular pressure; AXL, axial length; SE, spherical equivalence; CCT, central corneal thickness.

*Comparison was performed using t test.

^†^Comparison was performed using chi-squared test.

### RNFL and GCIPL thickness according to alcohol consumption

**Figure 1:**
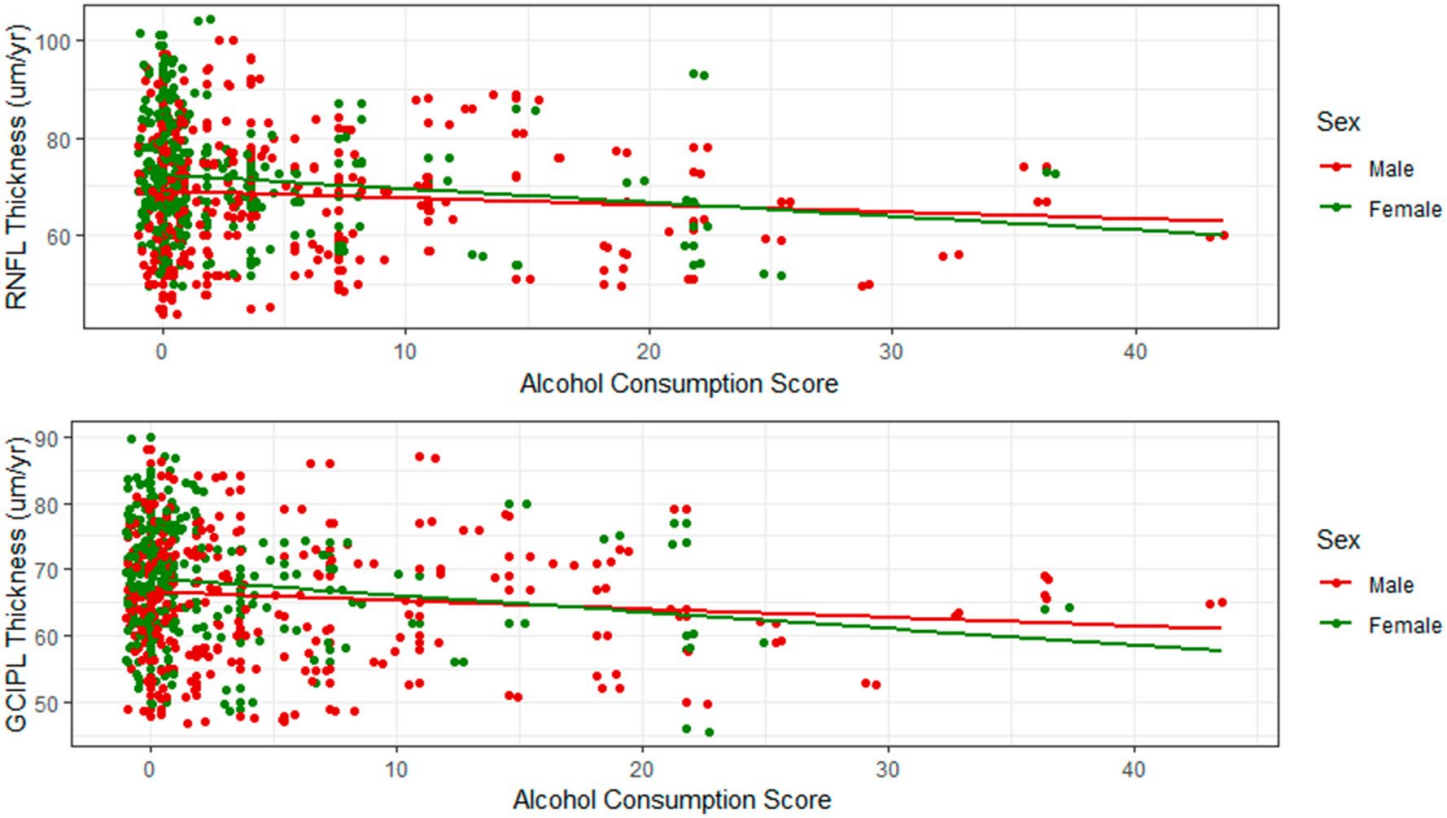
Correlation between Alcohol Consumption Score and Retinal Nerve Fiber Layer and Ganglion Cell-Inner Plexiform Layer Thicknesses. Pearson’s correlation test showed a significant negative correlation between alcohol consumption score and peripapillary retinal nerve fiber layer (RNFL) thickness (r = − 0.125, *P* = 0.008). In the subgroup analysis, only females reached marginal significance (r = − 0.126, *P* = 0.06); males did not (r = − 0.088, *P* = 0.19). Likewise, the alcohol consumption score demonstrated a significant negative correlation with macular ganglion cell-inner plexiform layer (GCIPL) thickness (r = − 0.139, *P* = 0.003). In the subgroup analysis, only females (r = − 0.150, *P* = 0.024), not males (r = − 0.104, *P* = 0.12), showed a significant negative correlation.

The peripapillary RNFL thickness tended to be thinner in the drinking group (69.0 ± 12.0 µm) than in the abstinence group (70.9 ± 11.5 µm) but with only marginal significance (*P* = 0.09). In the subgroup analysis, only eyes from the heavy drinking group (65.0 ± 10.9 µm) showed significantly thinner peripapillary RNFL thickness relative to those from the abstinence group (*P* = 0.023, Table 2). There was a significant negative correlation between alcohol consumption score and peripapillary RNFL thickness (*r* = –0.125, *P* = 0.008, Fig. 1). In the subgroup analysis, only females reached marginal significance (*r* = –0.126, *P* = 0.06); males did not (*r* = –0.088, *P* = 0.19). Binge drinkers tended to have thinner RNFL thickness than non-binge drinkers but without statistical significance (*P* = 0.23).

**Table 2:** Comparison of structural and functional parameters according to alcohol consumption.

	Abstinence group (*n* = 265)	Mild drinking group (*n* = 147)	Heavy drinking group (*n* = 33)	*P* value^†^	Post hoc analysis	Non-binge-drinking group (n = 141)	Binge drinking group (*n* = 39)	*P *value^†^	Post hoc analysis
RNFLT, μm	70.9 ± 11.5	69.9 ± 12.1	**65.0 ± 10.9**	**0.023**	**A > C**	69.8 ± 12.2	66.3 ± 11.0	0.06	
**GCIPLT, μm**	**68.1 ± 8.2**	**65.8 ± 9.6**	**63.8 ± 8.4**	**0.003**	**A > B, C**	**65.8 ± 9.5**	**63.9 ± 9.1**	**0.003**	**A > D, E**
MD, dB	− 8.3 ± 7.1	− 9.2 ± 7.9	− 10.4 ± 8.9	0.21		− 9.2 ± 7.9	− 10.4 ± 9.0	0.19	
PSD, dB	9.0 ± 4.7	9.4 ± 5.0	8.8 ± 4.8	0.64		9.4 ± 5.1	8.9 ± 4.4	0.77	
VFI, %	76.3 ± 21.3	73.8 ± 25.5	69.6 ± 27.7	0.25		74.0 ± 25.3	70.0 ± 27.8	0.23	

Mean ± standard deviation, statistically significant values are shown in bold.

RNFLT, retinal nerve fiber layer thickness; GCIPL, ganglion cell-inner plexiform layer; MD, mean deviation; PSD, pattern standard deviation; VFI, visual field index; A, abstinence group; B, mild drinking group; C, heavy drinking group; D, non-binge-drinking group; E, binge drinking group.

^†^Comparison was performed using one-way analysis of variance with post hoc Scheffe’s multiple comparison testing.

Macular GCIPL thickness was significantly thinner in the drinking group (65.5 ± 9.4 µm) than in the abstinence group (68.1 ± 8.2 µm, *P* = 0.002). Both mild drinking (65.8 ± 9.6 µm) and heavy drinking group eyes (63.8 ± 8.4 µm) had significantly thinner macular GCIPL thickness than did the abstinence group (*P* = 0.003, Table 2). Likewise, eyes of non-binge drinkers (65.8 ± 9.5 µm) as well as binge drinkers (63.9 ± 9.1 µm) had significantly thinner macular GCIPL thickness than did eyes of the abstinence group (*P* = 0.003, Table 2), though there were no significant differences between binge and non-binge drinkers. The alcohol consumption score showed a significant negative correlation with macular GCIPL thickness (*r* = –0.139, *P* = 0.003, Fig. 1). In the subgroup analysis, only females (*r* = –0.150, *P* = 0.024), not males (*r* = –0.104, *P* = 0.12), showed a significant negative correlation.

There were no significant differences in visual field parameters including mean deviation (MD), pattern standard deviation (PSD), and visual field index (VFI), between eyes from the abstinence and drinking groups (Table 2).

### RNFL and GCIPL thickness according to rs671 polymorphism

The minor allele frequency (MAF) for rs671 was 0.156. Of the 445 POAG patients, 322 were major homozygote (GG), 107 were heterozygote (GA), and 16 were minor homozygote (AA). However, there were no significant differences in peripapillary RNFL thickness, macular GCIPL thickness or visual field parameters (MD, PSD, or VFI), according to rs671 polymorphism (Table 3).

**Table 3:** Comparison of structural and functional parameters according to *ALDH2* rs671 polymorphism.

	ALDH2 rs671 genotype			*P* value
	GG (*n* = 322)	GA (*n* = 107)	AA (*n* = 16)	
RNFLT, μm	70.3 ± 12.0	69.6 ± 11.2	71.1 ± 10.5	0.83
GCIPLT, μm	67.4 ± 9.1	66.3 ± 8.2	66.0 ± 7.8	0.23
MD, dB	**− **8.6 ± 7.4	**− **8.8 ± 7.8	**− **10.2 ± 8.3	0.55
PSD, dB	9.2 ± 4.9	8.9 ± 4.5	9.4 ± 3.7	0.86
VFI, %	75.2 ± 22.8	75.0 ± 24.0	69.2 ± 28.7	0.52

Mean ± standard deviation, Comparison was performed using one-way analysis of variance with post hoc Scheffe’s multiple comparison testing.

RNFLT, retinal nerve fiber layer thickness; GCIPL, ganglion cell-inner plexiform layer; MD, mean deviation; PSD, pattern standard deviation; VFI, visual field index.

**Figure 2:**
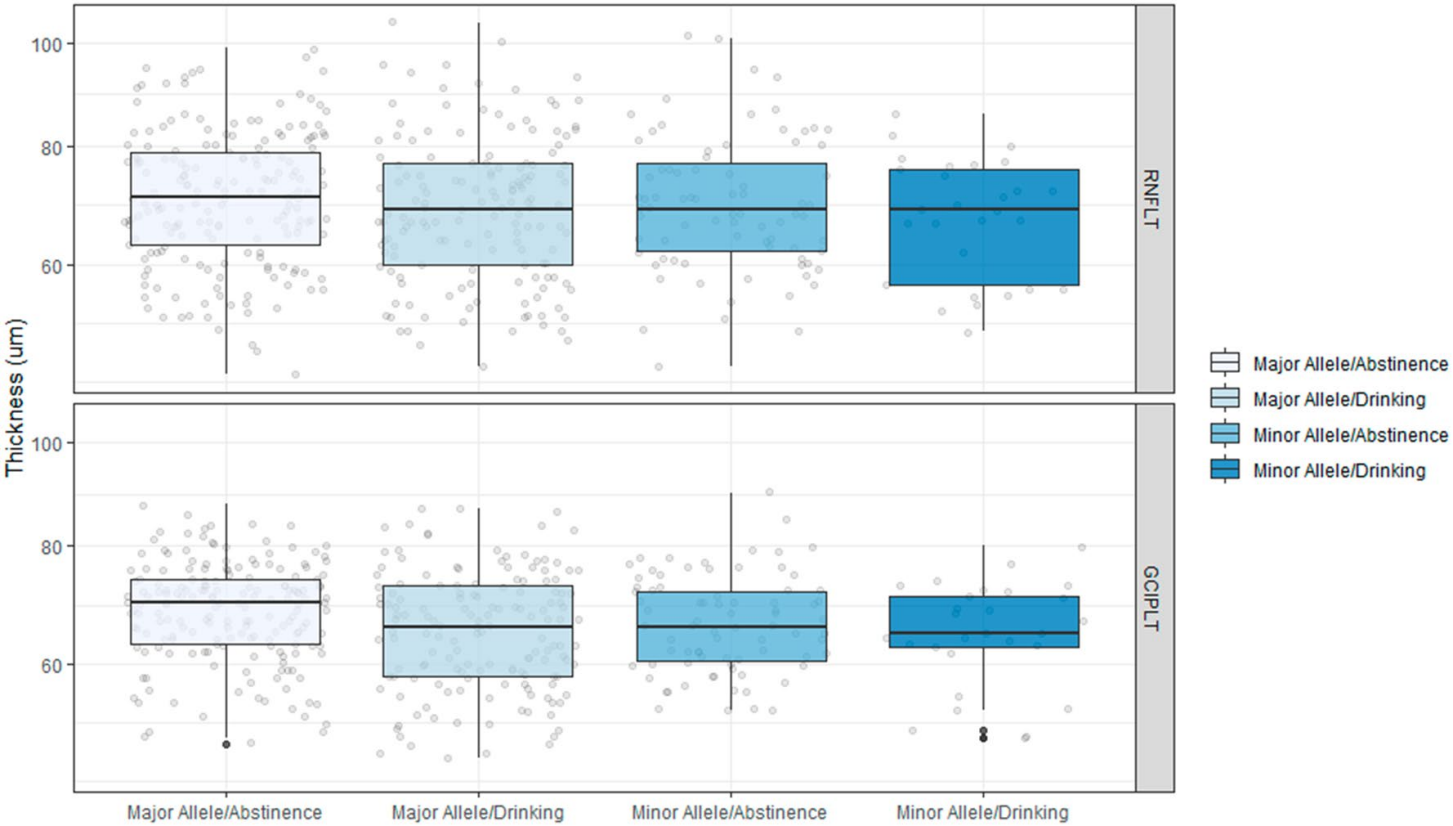
Retinal Nerve Fiber Layer Thickness and Ganglion Cell-Inner Plexiform Layer Thickness According to *ALDH2* rs671 Polymorphism and Alcohol Consumption. The peripapillary retinal nerve fiber layer (RNFL) and macular ganglion cell-inner plexiform layer (GCIPL) thicknesses were compared according to rs671 polymorphism and alcohol consumption. No significant differences were observed in peripapillary RNFL thickness. In terms of macular GCIPL thickness, eyes with the major allele for rs671 showed significantly thinner macular GCIPL thickness in the drinking group (65.5 ± 9.7 µm) than in the abstinence group (68.9 ± 8.1 µm, *P* = 0.009). The eyes with the minor allele for rs671 in both the abstinence (66.6 ± 8.0 µm) and drinking (65.1 ± 8.3 µm) groups tended to have thinner macular GCIPL thickness than did the major allele eyes in the abstinence group (68.9 ± 8.1 µm), but the difference was not statistically significant.

The study population was subdivided into four groups according to drinking status and rs671 polymorphism. In terms of peripapillary RNFL thickness, there was no significant difference among the subgroups (Fig. 2). In terms of macular GCIPL thickness, eyes with the major allele for rs671 showed significantly thinner macular GCIPL thickness in the drinking group (65.5 ± 9.7 µm) than in the abstinence group (68.9 ± 8.1 µm, *P* = 0.009, Fig. 2). The eyes with the minor allele for rs671 in both the abstinence (66.6 ± 8.0 µm) and drinking (65.1 ± 8.3 µm) groups tended to have thinner macular GCIPL thickness than did major allele eyes in the abstinence group (68.9 ± 8.1 µm), but the difference did not represent statistical significance.

### Factors associated with RNFL and GCIPL thickness

The factors associated with peripapillary RNFL and macular GCIPL thicknesses were investigated. In the univariate analysis, the factors associated with thinner peripapillary RNFL thickness were male sex (*P* = 0.002), greater baseline IOP (*P* < 0.001), greater vertical C/D (*P* < 0.001), smaller rim area (*P* < 0.001), greater alcohol consumption score (*P* = 0.008), and binge drinker (*P* = 0.029). The following variables that retained significance at *P* < 0.10 were included in the multivariable model: sex, baseline IOP, AXL, vertical C/D, rim area, alcohol consumption score, and binge drinker. To avoid interaction between alcohol consumption score and binge drinking, the GLM analysis was performed separately for these two variables. In multivariable model 1, sex (β = 1.754, *P* = 0.039), vertical C/D (β = –9.760, *P* = 0.043) and rim area (β = 36.286, *P* < 0.001) were significantly associated with thinner peripapillary RNFL thickness. In multivariable model 2, sex (β = 1.906, *P* = 0.026), vertical C/D (β = –9.718, *P* = 0.044) and rim area (β = 36.365, *P* < 0.001) were significantly associated with thinner peripapillary RNFL thickness (Table 4).

**Table 4:** Factors associated with RNFL thickness.

Variables	Univariate model			Multivariable model 1			Multivariable model 2		
	beta	SE	*P* value	beta	SE	*P* value	beta	SE	*P* value
Age, per year	− 0.063	0.042	0.13						
Sex, female	**3.395**	**1.099**	**0.002**	**1.754**	**0.848**	**0.039**	**1.906**	**0.855**	**0.026**
DM	− 1.317	1.515	0.38						
HTN	0.756	1.182	0.62						
Baseline IOP, per mmHg	**− 0.504**	**0.104**	** < 0.001**	0.008	0.082	0.92	0.015	0.082	0.85
AXL, per mm	− **0.729**	**0.374**	**0.05**	**− **0.098	0.269	0.71	**− **0.098	0.274	0.72
CCT, per μm	0.016	0.016	0.99						
Vertical C/D	− **52.566**	**4.213**	** < 0.001**	− **9.760**	**4.798**	**0.043**	**− 9.718**	**4.813**	**0.044**
Rim area, per mm^2^	**39.909**	**1.773**	** < 0.001**	**36.286**	**2.632**	** < 0.001**	**36.365**	**2.63**	** < 0.001**
Disc area, per mm^2^	1.087	1.173	0.35						
Smoking, per pack-year	− 0.180	0.103	0.18						
Alcohol consumption score	− **0.826**	**0.311**	**0.008**	− 0.421	0.264	0.11			
Binge drinker	− **1.853**	**0.847**	**0.029**				**− **0.456	0.686	0.51
rs671 polymorphism	− 0.713	1.299	0.58						

Statistical analysis was performed using the generalized linear model (GLM). Statistically significant values are shown in bold.

*Factors with *P* < 0.10 in the univariate analysis were included in the multivariable analysis.

The same analysis was performed for macular GCIPL thickness. In the univariate analysis, the factors associated with thinner macular GCIPL thickness were male sex (*P* = 0.012), greater baseline IOP (*P* < 0.001), greater AXL (*P* < 0.001), greater vertical C/D (*P* < 0.001), smaller rim area (*P* < 0.001), greater alcohol consumption score (*P* = 0.003), and binge drinker (*P* < 0.001). In multivariable model 1, baseline IOP (β = –0.174, *P* = 0.008), AXL (β = –1.204, *P* < 0.001), vertical C/D (β = –8.585, *P* = 0.026), rim area (β = 21.482, *P* < 0.001) and alcohol consumption score (β = –0.446, *P* = 0.035) were significantly associated with thinner macular GCIPL thickness. In multivariable model 2, baseline IOP (β = –0.164, *P* = 0.013), AXL (β = –1.177, *P* < 0.001), vertical C/D (β = –8.606, *P* = 0.026) and rim area (β = 21.531, *P* < 0.001) were significantly associated with thinner macular GCIPL thickness (Table 5).

**Table 5:** Factors associated with GCIPL thickness.

Variables	Univariate model			Multivariable model 1			Multivariable model 2		
	beta	SE	*P* value	beta	SE	*P* value	beta	SE	*P* value
Age, per year	0.021	0.031	0.50						
Sex, female	**2.093**	**0.829**	**0.012**	0.422	0.680	0.53	0.483	0.686	0.48
DM	− 0.701	1.140	0.53						
HTN	0.602	0.889	0.50						
Baseline IOP, per mmHg	**− 0.458**	**0.077**	** < 0.001**	**− 0.174**	**0.065**	**0.008**	**− 0.164**	**0.0658**	**0.013**
AXL, per mm	− **1.588**	**0.272**	** < 0.001**	− **1.204**	**0.215**	** < 0.001**	**− 1.177**	**0.220**	** < 0.001**
CCT, per μm	0.003	0.012	0.77						
Vertical C/D	− **33.181**	**3.330**	** < 0.001**	− **8.585**	**3.846**	**0.026**	**− 8.606**	**3.859**	**0.026**
Rim area, per mm^2^	**26.722**	**1.484**	** < 0.001**	**21.482**	**2.109**	** < 0.001**	**21.531**	**2.116**	** < 0.001**
Disc area, per mm^2^	0.473	0.883	0.59						
Smoking, per pack-year	− 0.170	0.077	0.13						
Alcohol consumption score	− **0.690**	**0.233**	**0.003**	− **0.446**	**0.212**	**0.035**			
Binge drinker	− **2.165**	**0.632**	** < 0.001**				**− **0.829	0.551	0.133
rs671 polymorphism	− 1.043	0.976	0.29						

Statistical analysis was performed using the generalized linear model (GLM). Statistically significant values are shown in bold.

*Factors with *P* < 0.10 in the univariate analysis were included in the multivariable analysis.

## Discussion

The present study found a negative correlation between alcohol consumption and macular GCIPL thickness, which association was more remarkable in females. Greater alcohol consumption score was significantly associated with thinner macular GCIPL thickness after controlling for confounding factors. However, the present study did not find any significant association of *ALDH2* rs671 polymorphism with glaucoma severity marked by peripapillary RNFL or macular GCIPL thickness.

Alcohol is primarily metabolized in the liver but is also metabolized in extrahepatic tissues such as the brain^21^. Alcohol is oxidized to acetaldehyde, a highly toxic byproduct that can cause tissue damage, by three enzymes: alcohol dehydrogenase (ADH), catalase, and cytochrome P450 isoenzymes^22^. During this process, reactive oxygen species (ROS) are also generated, making the neural tissues vulnerable to oxidative stress. Acetaldehyde is then further metabolized to acetate by mitochondrial aldehyde dehydrogenase2 (ALDH2). Since acetaldehyde is capable of binding to proteins (i.e. enzymes, microsomal proteins, and microtubules) and forming adducts with DNA, which ultimately results in impaired target-organ function^21,22^, ALDH2′s acetaldehyde-removal activity has attracted clinical interest.

The genetic diversity of *ALDH2*, on chromosome 12, is well established elsewhere^2,17,23–25^. The G-to-A missense mutation in exon 12, where glutamate at position 504 is replaced by lysine (Glu504Lys), is one of the representative SNPs (rs671) in *ALDH2*. Interestingly, the minor alleles of rs671 are very common in East Asians (being present in up to 50% of a given population), but less common in Caucasians^26,27^. The present study revealed an MAF for rs671 of 15.6%, which is quite consistent with previous findings from Korean populations^28,29^. The heterozygote and minor homozygotes for this SNP lead to decreased activity of ALDH2, affecting 17–35% and 1–3% of ALDH2 activities, respectively^30^. Increased acetaldehyde concentration after alcohol consumption has been further confirmed in subjects with rs671 minor alleles^31^.

It has been well documented that subjects with the rs671 minor allele are not likely to have alcohol dependence and more likely to be reluctant to drink, due to the acute effect of facial flushing or palpitation after alcohol consumption, which fact was also confirmed in the present study^32^. Contrary to our expectation, however, rs671 polymorphism did not affect the peripapillary RNFL or macular GCIPL thicknesses in POAG eyes. The present study demonstrated the tendency to thinner macular GCIPL thickness in eyes with the minor allele for rs671 than in eyes with the major alleles, but the difference was not statistically significant. The post hoc power was 0.999 for RNFL thickness (effect size *f* = 0.281, *α* error probability = 0.05, total sample size = 445) and 1.000 for GCIPL thickness (effect size *f* = 0.512, *α* error probability = 0.05, total sample size = 445). We could obtain sufficient power for the association analysis between rs671 polymorphism and POAG severity.

The present study found that greater alcohol consumption was significantly associated with thinner macular GCIPL thickness after compensation for several confounding factors including baseline IOP, AXL, and optic nerve head parameters. This is consistent with a recent large UK cohort study reporting that frequent alcohol consumption is associated with thinner macular RNFL, GCC and GCIPL^16^. Speculative explanations for the present finding are as follows. First, alcohol metabolism itself can aggravate oxidative stress in the central nervous system (CNS) as the result of increased production of ROS by catalase and cytochrome P450 isoenzymes^21,22^. Alcohol is mainly metabolized in the liver, but catalase has emerged as the main alcohol-metabolizing enzyme in the brain^33^. Cytochrome P450 2E1 (CYP2E1) also converts alcohol to acetaldehyde and generates ROS that cause neuronal injuries^34^. As the retina is one of the most energy-consuming organs for maintenance of vision, the retinal ganglion cells might be vulnerable to alcohol-induced oxidative stress. Second, chronic alcohol consumption can lead to thiamine deficiency due to inadequate nutritional intake, reduced uptake of thiamine from gastrointestinal tracts, or impaired utilization of thiamine in the cells^35^. Thiamine deficiency can cause impaired cerebral glucose metabolism, and exacerbate CNS dysfunction, a typical example being Wernicke encephalopathy. In line with this idea, previous reports have shown that thiamine levels in glaucoma patients are low^36^ and that thiamine protects against glaucoma^37^. The thiamine concentration associated with alcohol consumption in POAG patients needs to be further verified in future studies. Third, alcohol consumption can modify the gastrointestinal microbiome through the acute and chronic effects of alcohol on gastric motility dysfunction, changes in gastric acid output, and direct mucosal damage^38,39^. A recent study from Chen et al.^40^ raised the issue of the role of commensal microflora-induced T cell responses in glaucomatous progressive neurodegeneration. They postulated that pre-sensitized T cells that had been exposed to commensal microflora mediated glaucomatous neurodegeneration in response to IOP increase in mouse eyes. Recent evidence showing a significant association between Helicobacter pylori infection and open-angle glaucoma can further support this hypothesis^41,42^.

In the present study, the effect of alcohol consumption on macular GCIPL thickness was more remarkable in females than in males. This finding is consistent with those for African-American women^14^. Generally, females are likely to be more vulnerable to alcohol than males, as they have a lower proportion of body water than males, resulting in higher blood concentrations of alcohol after drinking^43^. ADH activity is also known to be lower in women than in men^44^. Taken together, the present findings indicate that excessive alcohol consumption can be more harmful in women than in men, in terms of its effects on glaucoma susceptibility.

The present study has several limitations. First, as it was based on a questionnaire for alcohol consumption, it was difficult to acquire the exact alcohol consumption of each participant. Thus, this study design might be limited in its suitability for investigation of the precise dose-dependent effects of alcohol on glaucoma severity. However, the relatively large number of subjects in the present study along with the alcohol consumption scoring system employed was helpful in revealing the negative relationship between alcohol consumption and macular GCIPL thickness. Second, as the present study was cross-sectional, it was difficult to evaluate the risk of excessive alcohol consumption with respect to the incidence or progression of glaucoma. Additional case–control or longitudinal cohort studies on alcohol consumption and glaucoma risk are essential to gain more practical information. Third, the present study recruited only POAG patients but not non-glaucomatous subjects. This may have caused the selection bias on the correlation analysis as the POAG eyes have thin RNFL and GCIPL that belong to the pathogenic end of the spectrum. However, the present findings still have clinical significance in warning POAG patients to avoid excessive alcohol consumption regardless of *ALDH2* rs671 polymorphism, as it was significantly associated with thinner macular GCIPL thickness. Lastly, the present study investigated only rs671 polymorphism but not the whole tag SNPs in *ALDH2*. Full sequencing of the *ALDH2* gene may reveal additional significant association results that deserve further exploration in the near future.

In conclusion, excessive alcohol consumption was negatively correlated with macular GCIPL thickness in POAG patients. *ALDH2* rs671 polymorphism, which is prevalent in East Asians, can alter ALDH2 activity during alcohol metabolism, but did not show any significant association with peripapillary RNFL or macular GCIPL thickness. However, it should be noted that subjects with the rs671 minor allele tend to drink less alcohol, which fact might be related to the present insignificant findings with regard to glaucoma severity. Our data suggest that glaucoma patients should avoid excessive alcohol consumption regardless of *ALDH2* polymorphism.

## Methods

The present study was initiated as a part of the GLAU-GENDISK (GLAUcoma GENeDIscovery Study in Korea) project, which is an ongoing prospective study designed in 2011^45^. The primary objective of the GLAU-GENDISK project is to investigate and identify novel genetic susceptibility loci for various types of glaucoma in a Korean population. The secondary objectives included establishing the genotype–phenotype relationships in glaucoma patients and constructing new disease prediction models. The present study was approved by the Seoul National University Hospital Institutional Review Board and followed the tenets of the Declaration of Helsinki (1964). The subjects who met the eligibility criteria provided written informed consent to participate.

### Study population

The detailed information for the GLAU-GENDISK study population is reported elsewhere^45^. The present study prospectively enrolled 551 POAG patients from Glaucoma Outpatient Clinics in Seoul National University Hospital. POAG was defined as the presence of glaucomatous optic disc changes with corresponding glaucomatous visual field (VF) defects and an open-angle confirmed by gonioscopy. Glaucomatous optic disc changes were defined as typical neuroretinal rim thinning, notching, excavation, or RNFL defects. Glaucomatous VF defects were defined as (1) glaucoma hemifield test values outside the normal limits, (2) three or more abnormal points with a probability of being normal of *P* < 5%, of which at least one point has a pattern deviation of *P* < 1%, or (3) a pattern standard deviation of *P* < 5%. The VF defects were confirmed on two consecutive reliable tests (fixation loss rate ≤ 20%, false-positive and false-negative error rates ≤ 25%).

The subjects underwent a complete ophthalmic examination, including a visual acuity assessment, slit-lamp biomicroscopy, intraocular pressure (IOP) measurement by Goldmann applanation tonometry, gonioscopy, refractions, dilated fundus examination, disc stereophotography, and red-free fundus photography using a digital fundus camera (VX-10; Kowa, Nagoya, Japan) and standard automated perimetry (Humphrey C 24–2 SITA-Standard visual field; Carl Zeiss Meditec, Inc., Dublin, CA, USA). The central corneal thickness (CCT; Pocket II; Quantel Medical, Clermont-Ferrand, France) and axial length (AXL; AXIS-II Ultrasonic Biometer; Quantel Medical S.A., Bozeman, MT, USA) were measured. A 200 × 200 optic disc cube scan and 200 × 200 macular cube scan were performed using Cirrus HD-OCT (Carl-Zeiss Meditec), and the average peripapillary RNFL and macular GCIPL thicknesses were measured with the built-in analysis algorithm (software version 6.0; Carl Zeiss Meditec).

All of the subjects were questioned regarding their status as a current smoker or non-smoker. Smoking history was expressed as the cumulative amount of smoking (‘pack-years’ = number of years as a smoker x number of cigarettes per day/20).

When the two eyes were eligible for inclusion in the study, the eye with worse visual field (VF) mean deviation (MD) was selected. The present study excluded patients with missing OCT (*n* = 32) or with poor OCT scan quality (signal strength < 6, *n* = 53), eyes with macular diseases (epiretinal membrane [*n* = 16], age-related macular degeneration [*n* = 1], and macular edema [*n* = 1]), or patients for whom genotyping had failed (*n* = 3). Finally, 445 eyes of 445 POAG patients were included in the present study.

### Survey on alcohol consumption

Alcohol consumption was investigated by questionnaire. The subjects were asked about the amount and frequency of drinking during the week. The amount of drinking was based on the traditional Korean liquor, Soju. One bottle of Soju (18% alcohol, 360 mL) contains 50.86 g of alcohol. The Centers for Disease Control and Prevention (CDC) and National Institute on Alcohol Abuse and Alcoholism (NIAAA)^46,47^ define excessive alcohol consumption (per occasion or per week) as follows: binge drinking (≥ 4 standard drinks per occasion for a woman, and ≥ 5 standard drinks per occasion for a man) or heavy drinking (≥ 8 standard drinks per week for a woman, and ≥ 15 standard drinks per week for a man), where a standard drink is defined as containing 14.0 g of pure alcohol. Based on these criteria, subjects were categorized into an abstinence group (those who reported never drinking), a non-heavy-drinking group (those who drink < 8 standard drinks per week for a woman, and < 15 standard drinks per week for a man), and a heavy drinking group (those who drink ≥ 8 standard drinks per week for a woman, and ≥ 15 standard drinks per week for a man). In terms of binge drinking, subjects who reported drinking were categorized into a non-binge-drinking group (< 4 standard drinks per occasion for a woman, and < 5 standard drinks per occasion for a man) or a binge-drinking group (≥ 4 standard drinks per occasion for a woman, and ≥ 5 standard drinks per occasion for a man). Additionally, the alcohol consumption score was recorded as the cumulative amount of drinking, as calculated by multiplying the amount of drinking (the number of standard drinks) by the frequency of alcohol consumption per week.

### SNP genotyping

The detailed information for the SNP genotyping is reported elsewhere^45^. Blood samples were collected from all of the subjects, and the genomic DNA was extracted. Among the 551 study samples, 309 were analyzed by exome chip (Illumina, Inc.; San Diego, CA, USA) analysis and 242 were analyzed by TaqMan assay (Applied Biosystems, Carlsbad, CA, USA). The exome-chip analysis utilized the Human Exome Bead-Chip 12v1-1 system (Illumina, Inc.), which included 244,651 markers focused on protein-altering variants. Details regarding SNP content and selection strategies can be found at the exome array design webpage (https://genome.sph.umich.edu/wiki/Exome_Chip_Design). Genotype calling was performed using Illumina’s GenTrain version 2.0 clustering algorithm with GenomeStudio software (V2011.1). Cluster boundaries were determined using Illumina’s standard cluster file. After additional visual inspection of SNPs with call rates of less than 0.99 and of those with minor allele frequencies (MAFs) less than 0.002, 244, and 552 of 244,651 (99.96%) attempted markers were successfully genotyped with call rates greater than 98% (average call rate: 99.92%).

The TaqMan assay (Applied Biosystems, Carlsbad, CA, USA) for the SNP rs671 was conducted by the following steps: 1) preparation of approximately 20 ng of purified genomic DNA; 2) preparation of a genotyping mixture consisting of 2X genotyping master mix, 20X SNP genotyping assay, DNAse-free water and template DNA, and 3) a polymerase chain reaction (PCR) entailing 40 cycles of denaturation and annealing/extension steps. When the PCR was completed, the genotypes of the DNA samples were analyzed on the ABI prism 7900HT sequence detection system (Applied Biosystems, Foster City, CA, USA).

### Statistical analysis

Continuous variables were compared among the three groups by one-way analysis of variance (ANOVA) with Scheffe’s post hoc analysis. Categorical variables were compared using a chi-square test. Pearson’s correlation test was used to identify the correlation between alcohol consumption score and peripapillary RNFL and macular GCIPL thicknesses, respectively. The generalized linear model (GLM) was used to determine the factors (i.e. age, sex, diabetes mellitus, hypertension, baseline IOP, AXL, spherical equivalence [SE], CCT, vertical cup-to-disc ratio (C/D), disc rim area, disc area, smoking history (pack-year), alcohol consumption score, and rs671 polymorphism) associated with average RNFL or macular GCIPL thickness, first with a univariate model, and then with a multivariable model that included the univariate model variables with *P* < 0.10. All of the statistical analyses were performed with R version 3.6.1. (available at: https://www.r-project.org; accessed April 2019). Except where stated otherwise, the data are presented as mean ± standard deviations, and the level of statistical significance was set at *P* < 0.05.

## Data Availability

The datasets generated and/or analyzed in the current study are available from the corresponding author on reasonable request. The sequencing dataset is held at Seoul National University Hospital (SNUH), access to it requiring approval of the Institutional Review Board of SNUH.
